# Reduced Hippocampal-Striatal Interactions during Formation of Durable Episodic Memories in Aging

**DOI:** 10.1093/cercor/bhab331

**Published:** 2021-09-28

**Authors:** Hedda T Ness, Line Folvik, Markus H Sneve, Didac Vidal-Piñeiro, Liisa Raud, Oliver M Geier, Lars Nyberg, Kristine B Walhovd, Anders M Fjell

**Affiliations:** Research Group for Lifespan Changes in Brain and Cognition, Department of Psychology, University of Oslo, Oslo 0373, Norway; Research Group for Lifespan Changes in Brain and Cognition, Department of Psychology, University of Oslo, Oslo 0373, Norway; Research Group for Lifespan Changes in Brain and Cognition, Department of Psychology, University of Oslo, Oslo 0373, Norway; Research Group for Lifespan Changes in Brain and Cognition, Department of Psychology, University of Oslo, Oslo 0373, Norway; Research Group for Lifespan Changes in Brain and Cognition, Department of Psychology, University of Oslo, Oslo 0373, Norway; Department of Diagnostic Physics, Oslo University Hospital, Oslo 0424, Norway; Research Group for Lifespan Changes in Brain and Cognition, Department of Psychology, University of Oslo, Oslo 0373, Norway; Department of Radiation Sciences, Radiology, Umeå University, 901 87 Umeå, Sweden; Department of Integrative Medical Biology, Umeå University, 901 87 Umeå, Sweden; Umeå Center for Functional Brain Imaging, Umeå University, 901 87 Umeå, Sweden; Research Group for Lifespan Changes in Brain and Cognition, Department of Psychology, University of Oslo, Oslo 0373, Norway; Department of Radiology and Nuclear Medicine, Oslo University Hospital, 0372 Oslo, Norway; Research Group for Lifespan Changes in Brain and Cognition, Department of Psychology, University of Oslo, Oslo 0373, Norway; Department of Radiology and Nuclear Medicine, Oslo University Hospital, 0372 Oslo, Norway

**Keywords:** aging, durable memory encoding, fMRI, functional connectivity, long-term memory

## Abstract

Encoding of durable episodic memories requires cross-talk between the hippocampus and multiple brain regions. Changes in these hippocampal interactions could contribute to age-related declines in the ability to form memories that can be retrieved after extended time intervals. Here we tested whether hippocampal–neocortical– and subcortical functional connectivity (FC) observed during encoding of durable episodic memories differed between younger and older adults. About 48 younger (20–38 years; 25 females) and 43 older (60–80 years; 25 females) adults were scanned with fMRI while performing an associative memory encoding task. Source memory was tested ~20 min and ~6 days postencoding. Associations recalled after 20 min but later forgotten were classified as transient, whereas memories retained after long delays were classified as durable. Results demonstrated that older adults showed a reduced ability to form durable memories and reduced hippocampal–caudate FC during encoding of durable memories. There was also a positive relationship between hippocampal–caudate FC and higher memory performance among the older adults. No reliable age group differences in durable memory–encoding activity or hippocampal–neocortical connectivity were observed. These results support the classic theory of striatal alterations as one cause of cognitive decline in aging and highlight that age-related changes in episodic memory extend beyond hippocampal–neocortical connections.

## Introduction

Some experiences become long-lasting, durable episodic memories while others are short-lived and transient. Although a topic of debate, evidence suggests that the rate of long-term forgetting increases with age (Elliott et al. 2014), and the brain foundation for this is unknown. A number of alterations, involving temporocortical (Salami et al. 2014; Gorbach et al. 2017) and frontostriatal (Buckner 2004) circuits, contribute to the general reduction of episodic memory function in aging (Nyberg et al. 2012). It is unknown whether these also can explain why the ability to form long-lasting memories (“durable memories”) tends to decline even more with age than the ability to form memories that are contained for a few hours or less (“transient memories”; Mary et al. 2013). The aim of the present study was to test whether changes in functional connectivity (FC) between hippocampus and associated brain regions at encoding contribute to the observed age-related reduction in ability to form durable episodic memories.

Increased activity in widespread cortical areas and in the hippocampus along with decreased activity in default mode network (DMN) regions is predictive of subsequent memory success after short time intervals in young adults (Kim 2011). For older adults, a pattern of relatively decreased activity in task-positive and increased activation in task-negative (default mode) networks has been observed during encoding (Maillet and Rajah 2014). Studies that have tested memory after more extended delays (days/weeks) suggest that partially independent mechanisms support the formation of durable versus transient memories. Durable memories were associated with greater encoding activity in inferior lateral parietal and posteromedial regions compared with transient memories, with older adults exhibiting lower posteromedial activity than younger adults (Vidal-Piñeiro et al. 2017).

Importantly, fMRI studies have shown that memory durability is associated with factors beyond regional activity levels during encoding. While early regional activity levels likely promote rapid consolidation processes at the cellular level (Dudai 2004), hippocampal–neocortical communication appears critical in supporting the transformation of initial into long-lasting memories (Sneve et al. 2015). Systems consolidation is thought to rely on reactivation of initially stored representations through hippocampal–neocortical communication during postencoding periods of rest and sleep (Diekelmann and Born 2010; Stickgold and Walker 2013). Brain regions that are synchronously engaged during encoding, such as hippocampus and perceptual areas, continue to exhibit correlated activity in postencoding periods, and this FC predicts subsequent memory (Tambini et al. 2010; de Voogd et al. 2016; Tambini and Davachi 2019). One possibility is that such early FC indexes or tags memories that will undergo further postencoding systems consolidation (Diekelmann et al. 2009).

Compared with younger adults, older adults show reduced hippocampal–neocortical FC during encoding of episodic memories retrieved after shorter delays (Leshikar et al. 2010; Salami et al. 2014). Communication between hippocampus and dorsal striatum during encoding has also been shown to predict memory success (Sadeh et al. 2011). Further, older adults with greater dopamine (D2) receptor availability in hippocampus and caudate accompanied by higher resting-state FC between these regions have better episodic memory (Nyberg et al. 2016). This is interesting in the context of durable memories, as both hippocampus and striatum have been implicated in consolidation of memory (the ventral striatum in motor sequence memory, see Albouy et al. 2008). Thus, an important question regards whether differences in FC between hippocampus and other subcortical regions such as the striatum during encoding can contribute to explain age-related differences in durable memory function. The specific aims of the present study were to examine whether older adults show less hippocampal–neocortical and hippocampal–subcortical FC than younger adults during encoding of durable memories and to test whether differences in FC predict memory performance. In the following, we approach these questions using generalized psychophysiological interaction (PPI) analyses (McLaren et al. 2012) on encoding data from a novel explicit associative memory task collected in groups of younger and older adults. We used a data-driven approach in which the hippocampal seed region and subcortical regions of interest (ROIs) for the FC analyses were defined based on where significant univariate effects (“activity”) were found. Encoding trials were classified into short- or long-lived (durable) episodic memories based on subsequent memory testing at different intervals, and the associated FC contrasted using PPIs. Associative or relational memory is known to be more vulnerable to aging than memory for single items (Old and Naveh-Benjamin 2008) and tends to engage the hippocampus to a greater extent than item memory (Davachi 2006). The current memory task was further development of a previously used associative memory task from our lab (see e.g., Sneve et al. 2015) and part of a larger research project designed to address several research questions (Age Consolidate Project 2017; https://cordis.europa.eu/project/id/725025). Unlike the previous task from our lab, the current encoding task was intentional and participants knew in advance that their memory would be tested several times after encoding. Repeated testing of the same memories allowed us to track and test each memory’s durability. Further, while the previous task required the participant to form an action-item association, the present task instead required the formation of face-item and place-item associations, which presumably would yield better experimental control over the associations.

## Materials and Methods

### Participants

About 48 younger adults aged 20–38 (mean (*M*) *=* 26.40, standard deviation (SD) = 4.19, 25 females) and 43 older adults aged 60–80 (*M =* 67.25, SD = 5.71, 25 females) participated in the study. All participants gave written informed consent, and the study was approved by the Regional Ethical Committee of South East Norway. Participants were required to be right-handed, speak Norwegian fluently and have normal or corrected-to-normal hearing and vision. None of the participants had a history of neurological or severe psychiatric disorders or serious head trauma, were under psychiatric treatment, or used any medicines known to affect nervous system functioning. Participants were further required to score ≥ 26 on the Mini Mental State Examination (MMSE; Folstein et al. 1975) and to obtain a normal IQ or above (IQ ≥ 85) score on the Wechsler Abbreviated Scale of Intelligence (WASI; Wechsler 1999). Younger adults had to score ≤ 16 on the Beck Depression Inventory (BDI; Beck et al. 1988) and older adults had to score ≤ 9 on the Geriatric Depression Scale (GDS; Yesavage et al. 1982; see Table 1 for participant characteristics). Participants were compensated for their participation.

**Table 1:** Participant characteristics

	Younger adults			Older adults		
	Mean (SD)	*n*	Range	Mean (SD)	*n*	Range
Age	26.40 (4.19)	48	20–38	67.25 (5.71)	43	60–80
Sex (female/male)	–	25/23	–	–	25/18	–
MMSE	29.19 (1.30)	48	27–30	28.85 (1.07)	42^a^	27–30
IQ	110.66 (9.06)	47^a^	90–125	120.55 (10.40)	42^a^	96–146
Education^b^	15.13 (1.98)	46^c^	13–18	16.24 (2.10)	41^c^	10–21
Depression	3.66 (2.31)	46^d^	0–20.50^e^	2.65 (3.24)	38^f^^,^^g^	0–16^h^
Interval between encoding and long-delay memory test (days)	6.64 (2.50)	46^i^	5–21^j^	6.10 (1.70)	43	5–13^j^

*Note:* Abbreviations: n, sample size.

^a^MMSE and IQ scores were missing for one older participant and IQ score was missing for one younger participant who could not complete testing due to COVID-19 restrictions.

^b^Education = Years of total education rounded down to the closest whole number.

^c^Education scores were missing for two younger participants and for two older participants.

^d^BDI scores were missing for two (younger) participants.

^e^One participant scored 19 and one scored 20.5 on BDI.

^f^Three older participants were wrongly given the BDI questionnaire instead of GDS. All three scored ≤5 on the BDI.

^g^Lack of GDS from 5 (older) participants (in which three of these completed BDI instead).

^h^One participant scored 16 on GDS.

^i^Two younger participants did not complete any of the long-delay memory tests.

^j^A Welch’s independent samples t-test (two-tailed) showed that there was not a significant difference in encoding long-delay interval between the younger (*M* = 6.64, SD = 2.50) and older participants (*M* = 6.10, SD = 1.70; *t*(79.55) = −1.1917, *P* = 0.2369)

The analyzed data in this study were drawn from a larger project that included multiple phases and tests (Age Consolidate Project 2017). Most participants (*N* = 75) completed the fMRI paradigm twice, over two separate “visits,” separated by a minimum of 6 days. The time of day for encoding and the short-delay memory test was counterbalanced within-subjects, with one encoding and test session completed in the evening in one visit and one encoding and test session completed in the morning during the other visit.

### fMRI Task

Stimulus material (from both visits combined) consisted of a total of 256 real-life images of nonanimate everyday items, eight images of faces and eight images of places, as well as 256 auditory stimuli in the form of a prerecorded (female voice) name for each item. All item/auditory stimuli were two-syllable Norwegian words. A predetermined half of the stimuli (item images and corresponding auditory item-names 1–128, face images 1–4, and place images 1–4) constituted the task material for participants’ first visit, and the other half of the stimuli constituted the task material for participants’ second visit (i.e., item images and corresponding auditory item-names 129–256, face images 5–8, and place images 5–8). Apart from the specific images used, the tasks were identical across visits. Training task stimuli consisted of 16 cartoon images and item-names from the same stimuli categories (i.e., items, faces, and places). Item images were obtained mainly from the Bank of Standardized Stimuli (Brodeur et al. 2010), some from StickPNG.com and from Google Advanced Image Search under the license “labelled for reuse with modification.” Face images were obtained from Oslo Face Database (described in Chelnokova et al. 2014). Tasks were designed and displayed using MATLAB 9.7.0 and Psychtoolbox-3 3.0.16.

### Experimental Design

**Figure 1:**
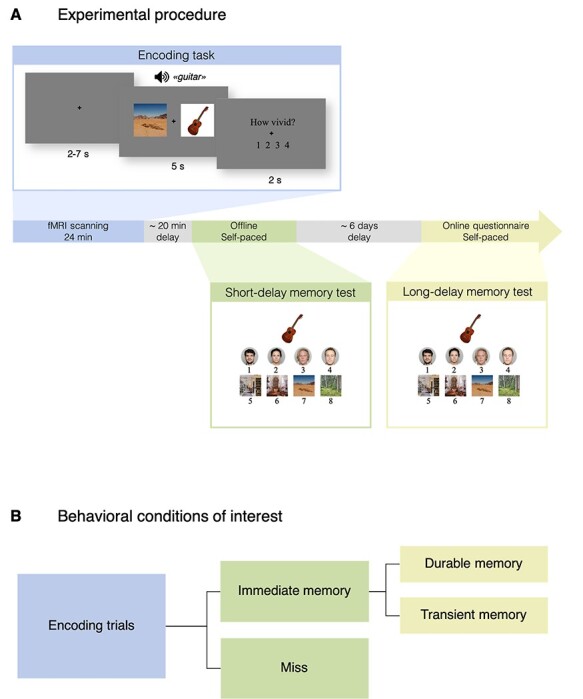
Experimental paradigm. (*A*) Schematic depiction of the experimental procedure. Participants encoded item-face/place associations during fMRI scanning (blue). Immediately after scanning and approximately 20 min after the end of the last encoding run, participants’ memory for associations were tested using an eight alternative forced-choice (8AFC) source memory test (green; “Immediate memory test”). Participants’ memory was tested again after an extended delay of approximately 6 days (yellow; “Long-delay memory test”). Note: The face images presented here are not identical to those used in the task, but a selection of face images in which the individuals depicted have consented to their faces being publicly displayed (from the Oslo Face Database; Chelnokova et al. 2014). (*B*) Schematic of the behavioral conditions of interest: Immediate memory included those items that were correctly linked with their face- or place associate at the short-delay test, regardless of long-delay memory and miss included those items that were not correctly linked with their face- or place associate at the short-delay 8AFC test, regardless of long-delay memory. We divided the pool of immediate memory trials into two further conditions of interest based on whether item-face/place associations were also remembered after a longer delay: 1) durable memory included those items that were correctly linked with their face- or place associate at both the immediate memory test and the long-delay test, while 2) transient memory included those items that were correctly linked with their face- or place associate at the immediate memory test but not the long-delay test.

The experimental paradigm consisted of three parts in this study: 1) Encoding of item-face/place pairs was performed during scanning, 2) a source memory test was completed outside the scanner immediately after the scan session (~20 min after encoding), and 3) another source memory test was completed ~6 days after this (see Fig. 1*A* for a schematic depiction of the experimental procedure). Of note, as part of a larger project, participants also completed two memory tests in between the 20-min and the long-delay (~6 days) test, approximately ~12 h after the encoding task. Data from these (12-h delayed) tests were not included in the present study. However, it means that participants’ memory was tested three times before the final, long-delay memory test (see Discussion for a discussion of the potential issues of repeated recall). Participants were informed that they would complete an association task in the scanner followed by several memory tests after scanning. Prior to entering the scanner, participants completed a training task, using the same two-button response grips to respond as in the scanner.

For the fMRI encoding task, participants were instructed to vividly imagine an interaction between the item and the face or place while presented on the screen, and afterwards to rate the vividness of their imagination on a scale from 1 to 4, in which 1 was “not vivid at all” and 4 was “very vivid.” The task consisted of two consecutively administered runs, with 64 trials in each run (note: when participants completed the fMRI encoding task twice over two separate visits, task data constituted 2 × 2 runs of 64 trials, i.e., 256 trials altogether). The pairing of an item and a face/place was randomly generated and unique for each participant. All visual stimuli were presented on a gray background and text appeared in black color. The item and face/place were presented on screen for 5 s while participants concurrently heard the name of the item three times (e.g., “donut,” “donut,” and “donut”). Of note, auditory presentations of item names were included in the fMRI task as this study was a part of a larger project in which the auditory stimuli serve a specific purpose. Then, the question “How vivid?” with numbers 1–4 below appeared on the screen for 2 s, which was the time window participants had to produce a response. After 2 s, the question was replaced by a fixation cross that remained on the screen until the beginning of the next trial, and variable intertrial intervals (ITIs; 2–7 s) were used. The order of stimuli conditions and ITI’s were optimized using optseq2 (https://surfer.nmr.mgh.harvard.edu/optseq/).

Immediately after scanning and approximately 20 min after the end of the last encoding run, participants practiced and completed a computerized eight alternative forced-choice (8AFC) memory test outside the scanner (immediate source memory test). The 8AFC test included all the items from the encoding task, which one-by-one were presented in the middle of the screen, together with the four alternative faces (above) and the four alternative places (below). Participants were instructed to select the alternative that had been presented together with each item during encoding. The 8AFC test was self-paced. After a response had been made, the item and selected alternative remained on the screen for 1 s, followed by a 1-s fixation interval before the start of the next trial. Participants additionally completed a similar 8AFC memory test via an online questionnaire ~6 days later (long-delay source memory test). The online memory test was made available to the participants 5 days postencoding, and participants were instructed to complete the test as soon as possible. Two participants did not complete the delayed test and were excluded from all analyses reflecting durable memory performance. About 85 participants completed the test within 5–8 days postencoding (see Table 1). The remaining four participants (two older, two younger adults) returned the tests 12–21 days postencoding. Due to the higher delay interval experienced by these four participants, all analyses concerning durable memory performance were also run on the *N* = 85 version of the dataset, that is, excluding participants with delay intervals >8 days.

Task data from both visits were collapsed and included in analyses. All 91 participants completed the fMRI encoding task and the short-delay memory test at least once. Two younger participants did not complete any of the long-delay memory tests and were thus discarded from the behavioral analysis of the long-delay performance (46 younger adults remaining; 20–38 years, *M =* 26.57, SD = 4.19, 25 females). For the behavioral analyses, test trial responses were classified as follows: 1) Immediate memory, consisting of items that were correctly linked with their face- or place associate in the immediate 8AFC test, and 2) durable memory, consisting of items that were correctly linked with their face- or place associate in both the immediate and delayed 8AFC test. That is, the immediate memory condition was based on performance on the first (immediate) test only and regardless of long-delay memory, while the durable memory condition was based on performance on both the immediate and long-delay memory tests combined (see Fig. 1*B* for a schematic of all the behavioral conditions of interest including two additional fMRI behavioral conditions that are described in the fMRI activity analysis section).

### MRI Acquisition

Imaging data were collected on a 3 T Siemens Prisma MRI system using a 32-channel Siemens head coil (Siemens Medical Solutions, Erlangen, Germany) at Rikshospitalet, Oslo University Hospital. The functional parameters were equivalent across all fMRI runs: 56 transversally oriented slices (no gap) were measured using a BOLD-sensitive T2^*^-weighted Echo Planar Imaging (EPI) sequence (TR = 1000 ms; TE = 30 ms; flip angle = 63°; matrix = 90 × 90; voxel size = 2.5 × 2.5 × 2.5 mm^3^; FOV = 225 × 225 mm^2^; interleaved acquisition; multiband factor = 4, phase encoding direction = AP). Each encoding run produced 724 volumes. At the start of each fMRI run, 10 dummy volumes were collected to avoid T1 saturation effects in the analyzed data. Anatomical T1-weighted (T1w) MPRAGE images consisting of 208 sagittally oriented slices (TR = 2400 ms; TE = 2.22 ms; TI = 1000 ms; flip angle = 8°; matrix = 320 × 300 × 208; Grappa factor = 2; voxel size = 0.8 × 0.8 × 0.8 mm^3^; FOV = 256 mm ×240 mm) and T2-weighted (T2w) SPACE images consisting of 320 sagittally oriented slices (TR = 3200 ms; TE = 5.63 ms; matrix = 320 × 300 × 208; voxel size = 0.8 × 0.8 × 0.8 mm^3^; FOV = 256 mm × 240 mm) were also obtained. Additionally, spin-echo field map sequences with opposing phase encoding directions (anterior–posterior and posterior–anterior) were acquired for distortion correction of the EPI images. Visual stimuli were presented on a 32-inch LCD monitor (NordicNeuroLab, Bergen, Norway) that was seen by participants through a tilted mirror, which was attached to the coil. Participants responded by using the ResponseGrip system (NordicNeuroLab, Bergen, Norway). Auditory stimuli were presented with the OptoActive noise canceling (ANC) II™ headphones (Optoacoustics Ltd, Israel, http://www.optoacoustics.com/).

### Preprocessing

The Python module Pydeface (Gulban et al. 2019) was used to remove facial structure from MRI images. Data were organized and named according to the BIDS (Brain Imaging Dataset Specification; Gorgolewski et al. 2016). Results included in this manuscript come from preprocessing performed using FMRIPREP version 1.5.3 (Esteban et al. 2019, 2020), a Nipype (Gorgolewski et al. 2011, 2017) based tool. Each T1w volume was corrected for INU (intensity nonuniformity) using N4BiasFieldCorrection v2.1.0 (Tustison et al. 2010) and skull-stripped using antsBrainExtraction.sh v2.1.0 (using the OASIS template). Brain surfaces were reconstructed using recon-all from FreeSurfer v6.0.0 (Dale et al. 1999), and the brain mask estimated previously was refined with a custom variation of the method to reconcile ANTs-derived and FreeSurfer-derived segmentations of the cortical gray matter of Mindboggle (Klein et al. 2017). T2w volumes were used to improve pial surfaces with recon-all. Spatial normalization to the ICBM 152 Nonlinear Asymmetrical template version 2009c (Fonov et al. 2009) was performed through nonlinear registration with the antsRegistration tool of ANTs v2.1.0 (Avants et al. 2008), using brain-extracted versions of both T1w volume and template. Brain tissue segmentation of cerebrospinal fluid (CSF), white matter (WM), and gray matter (GM) was performed on the brain-extracted T1w using FAST (Zhang et al. 2001; FSL v5.0.9). Functional data were slice time corrected using 3dTshift from AFNI v16.2.07 (Cox 1996) and motion corrected using MCFLIRT (FSL v5.0.9; Jenkinson et al. 2002). Distortion correction was performed using a custom implementation (https://github.com/markushs/sdcflows/tree/topup_mod) of the TOPUP technique (Andersson et al. 2003). This was followed by co-registration to the corresponding T1w using boundary-based registration (Greve and Fischl 2009) with six degrees of freedom, using bbregister (FreeSurfer v6.0.0). Motion correcting transformations, field distortion correcting warp, BOLD-to-T1w transformation, and T1w-to-template (MNI) warp were concatenated and applied in a single step using antsApplyTransforms (ANTs v2.1.0) with Lanczos interpolation. Frame-wise displacement (Power et al. 2014) was calculated for each functional run using the implementation of Nipype. ICA-based Automatic Removal Of Motion Artifacts (AROMA) was used to separate data into signal and noise components (Pruim et al. 2015). Many internal operations of FMRIPREP use Nilearn (Abraham et al. 2014), principally within the BOLD-processing workflow. For more details of the pipeline, see https://fmriprep.readthedocs.io/en/latest/workflows.html. The visual reports automatically generated by FMRIPREP per participant were manually inspected to ensure sufficient quality of the preprocessed data. Quality control of the structural data was performed using MRIQC (Esteban et al. 2017).

fMRI data were denoised prior to statistical analysis. First, nonaggressive removal of AROMA-classified noise components was carried out using the fsl_regfilt command-line tool. Average WM- and CSF signal time series (eroded masks) were extracted from the AROMA-denoised data. Then, Nilearn (function *clean_img*) was used for detrending, temporal high-pass filtering (0.008 Hz) and regression of WM and CSF time series from the AROMA-denoised data together with six estimated motion parameters. Finally, the mean signal was added back to the denoised data. Spatial smoothing (6 mm FWHM) was performed using FSL SUSAN in volume space and Freesurfer’s mri_surf2surf in surface space.

### Statistical Analysis

#### Behavioral Analysis

In order to test for age differences in immediate and durable memory, two Welch’s independent samples *t*-tests (two-tailed), comparing memory performance in the immediate memory condition and in the durable memory condition between the younger and older adults, were conducted. To determine whether there was a unique age difference in durable memory, an analysis of covariance (ANCOVA) was performed using short-delay memory performance as a covariate. As we expected a high correlation between short delay and durable memory performance, it was important to control for the short-term performance level when assessing unique age differences in durable memory. Statistical significance was considered at *P* < 0.05. All 91 participants were included in the analysis of short-delay memory performance, while the 89 of these who completed the long-delay test were included in the analysis of long-delay memory performance.

#### fMRI Activity Analysis

##### First-level GLM design

For the fMRI activity analyses, first-level general linear models (GLM) were set up, consisting of the conditions of interest modeled as events with onsets and durations corresponding to the item encoding period (5 s) and convolved with a two-gamma canonical hemodynamic response function (HRF) with temporal and dispersion derivatives. Each of the 256 encoding items was assigned to one or several conditions based on the participant’s response to the item at test, and defined as follows: 1) the immediate memory condition consisted of items that were correctly linked with their face- or place associate at the short-delay 8AFC test; 2) the miss condition consisted of items that were not correctly linked with their face- or place associate at the short-delay 8AFC test; 3) the durable memory condition consisted of items that were correctly linked with their face- or place associate both in the short- and long-delay 8AFC tests; and 4) the transient memory condition consisted of items that were correctly linked with their face- or place associate in the short-delay 8AFC test but not in the long-delay 8AFC test (see Fig. 1*B* for a schematic of the behavioral conditions of interest). Thus, the immediate memory and miss conditions were based on retrieval data from the first (immediate) test only and regardless of later memory, while the durable- and transient memory conditions were based on retrieval data from both the immediate- and long-delay tests. While most studies have tested participants’ memory only after short or immediate delays, our experimental paradigm allowed us to further divide immediate memory trials into two distinct categories, namely those that were remembered (durable memory) and those that were not remembered (transient memory) several days later. Hence there was no overlap between the durable- and transient memory conditions. Two models were set up: one considering performance on the immediate memory test that included the immediate- and miss condition regressors, and one model considering performance on the long-delay test that included the durable-, transient, and miss conditions. As participants’ neural responses to face- and place stimuli likely modulated the BOLD signal, we included this as an additional parametric regressor in both models (+1 for faces, −1 for places) to capture BOLD variance associated with face and place stimuli. However, as the responses to faces/places were not of main interest to our research questions, this regressor was not included in any of the contrasts. We defined two contrasts of interest, namely the durable memory contrast (durable versus transient memory conditions) and the immediate memory contrast (immediate versus miss conditions). Inclusion of the miss condition in the second model considering long-delay performance was to capture BOLD variance associated with miss trials, and this condition was not included in the durable memory contrast. Importantly, the immediate memory contrast was equivalent to the type of contrast used in most studies on subsequent memory, in which memories are tested after a short delay and only once. This type of contrast thus contains a mix of memories that are transient and memories that become durable. Testing the same memories twice allowed us to investigate whether a memory was also remembered after an extended interval or only transiently (i.e., the durable memory contrast).

##### Univariate activity analysis

For each individual, we computed the durable and the immediate memory contrasts based on the parameter estimates for further statistical analysis. Next, individual durable and immediate memory contrasts were brought to group level ordinary least square GLM analysis with sex included as a covariate. We tested the main effects of durable and immediate memory across all participants with a sufficient number of trials in each memory condition (see end of this paragraph for exclusion criteria and sample descriptions for the different memory contrasts). A second GLM, also with sex included as a covariate, was used to test the effects of age on the durable/immediate memory contrasts. Statistical significance was tested at each cortical vertex, and the resulting statistical estimates were corrected for multiple comparisons using a cluster extent-based approach: Vertices were thresholded at *P* < 0.001 and the remaining clusters tested through permutation inference across 5000 iterations using PALM. Cluster significance was considered at a family-wise error (FWE)-corrected level of *P* < 0.05. In addition to the surface-based cortical analysis, individual contrast estimates were extracted from subcortical ROIs. These ROIs were defined by extracting participants’ MNI152-transformed FreeSurfer automatic subcortical segmentation (aseg) masks and creating a “pruned” mask containing only voxels assigned to the same aseg structure in all participants (corresponding to a within-sample probabilistic threshold of 1.0). Statistical significance was tested through permutation inference across 5000 iterations on the data contained in the pruned mask using LISA, a nonparametric and threshold-free framework that applies a nonlinear, edge-preserving spatial filter to the permuted z-maps (Lohmann et al. 2017). Multiple comparison correction is achieved by a voxel-wise control of the false discovery rate (FDR) in the filtered maps. LISA has been shown to improve statistical power and sensitivity in detecting small activation clusters (Lohmann et al. 2018). Statistical significance was considered at an FDR-corrected level of *P* < 0.05. Participants with <8 trials in any of the relevant conditions (i.e., immediate memory-, miss-, durable memory-, or transient memory conditions) were excluded from analysis (see Supplementary Table 1 in Supplementary Materials for average trial numbers, ranges, and percentiles in each memory condition for each age group). In order to ensure that the analyzed source memory conditions were dominated by trials reflecting true successful memory encoding, that is, to be certain that they would not be dominated by guessing (guessing rate = ⅛ or 12.5% accuracy), participants with a mean accuracy below 25% on the short-delay 8AFC test were also excluded. For the immediate memory contrast, 47 younger adults (20–36 years, *M =* 67.23, SD = 5.84, 24 females) and 41 older adults (60–80 years, *M =* 26.16, SD = 3.86, 25 females) were included in analysis. For the durable memory contrast, 45 younger adults (20–36 years, *M =* 26.31, SD = 3.87, 23 females) and 40 older adults (60–80 years, *M =* 67.36, SD = 5.85, 25 females) were included in analysis (see also Supplementary Table 1 in Supplementary Materials for sample sizes in each memory condition for each age group).

##### Psychophysiological interactions analysis

Finally, a generalized psychophysiological interaction analysis (gPPI) was performed in order to examine age differences in task-specific hippocampal connectivity to the neocortex and the subcortical ROIs using the gPPI toolbox (McLaren et al. 2012). Neocortex constituted the FreeSurfer-defined cortical surface. Subcortical ROIs were defined based on the results from univariate activity analyses. Specifically, two dorsal striatal ROIs were defined from the MNI152-transformed aseg masks confined to the right and left putamen and the right and left caudate. A hippocampal seed mask was functionally defined by selecting the significant left anterior hippocampal voxels from the durable memory (durable memory > transient memory) activity contrast within the MNI152-transformed hippocampal aseg mask common across all participants (see Fig. 4*A*). BOLD data (average time series) for the hippocampal seed region was deconvolved into estimates of neural events (Gitelman et al. 2003). Each task time course from the first-level activity GLM design matrix, representing the two stimulus conditions of the experimental design (durable memory and transient memory), was multiplied separately by the deconvolved neural estimates from the hippocampal seed region and convolved with a canonical HRF, creating PPI terms. First-level GLM’s were set up for each participant and included: 1) the PPI regressors for each task condition, 2) the observed BOLD signal in the hippocampal seed region, and 3) the original HRF-convolved task regressors, which were regressed onto volume ROI- and cortical surface data. To compare hippocampal task-specific connectivity between younger and older adults, the durable-transient hippocampal connectivity interaction was brought to group level ordinary least square GLM analysis, with sex included as a covariate. Significance was considered after multiple comparison corrections with the same PALM- and LISA routines as for the group activity analyses, for cortical surface- and volume ROI data, respectively, at *P* < 0.05 (FWE-corrected for PALM, FDR-corrected for LISA). In order to examine the relationships between FC and durable memory performance, correlational analyses were carried out between PPI effects and the durable memory scores for both age groups using robust correlations (Shepherd's pi), as implemented in Pingouin 0.3.7 for Python (Vallat 2018). Following recommendations in Schwarzkopf et al. 2012, all reported *P* values associated with the robust correlation coefficients have been multiplied with 2 to ensure nominal false-positive rates at *alpha*-level 0.05. Fisher’s *z* was used to test the significance of the difference between these correlation coefficients using Fisher’s *r*-to-*z*-transformation. All reported *P* values are two-tailed. Based on recommendations from the existing literature, participants with less than 30 trials in either of the two relevant conditions (i.e., durable or transient memory conditions) were excluded from analysis in order to ensure a sufficient amount of trials per condition for PPI analyses (Huettel and McCarthy 2001; McLaren et al. 2012). About 30 younger adults (20–36 years, *M =* 26.68, SD = 4.23, 18 females) and 30 older adults (60–80 years, *M =* 67.8, SD = 6.14, 23 females) were included in the PPI analysis (see also Supplementary Table 1 in Supplementary materials for sample sizes in each memory condition for each age group).

#### Control Analyses

In order to evaluate the possible confounding effects of head motion and brain morphology on PPI results, we performed some additional control analyses. First, head motion (i.e., mean frame-wise displacement), intracranial volume (ICV), and hippocampal volume (left hemisphere, ICV-corrected) were compared in younger adults using Welch’s independent samples t-tests (two-tailed). Second, we repeated the GLM used to test the effects of age on the durable/immediate memory contrasts and the correlational analyses with head motion, intracranial and hippocampal volume included as covariates of no interest.

## Results

### Behavioral Results

**Figure 2:**
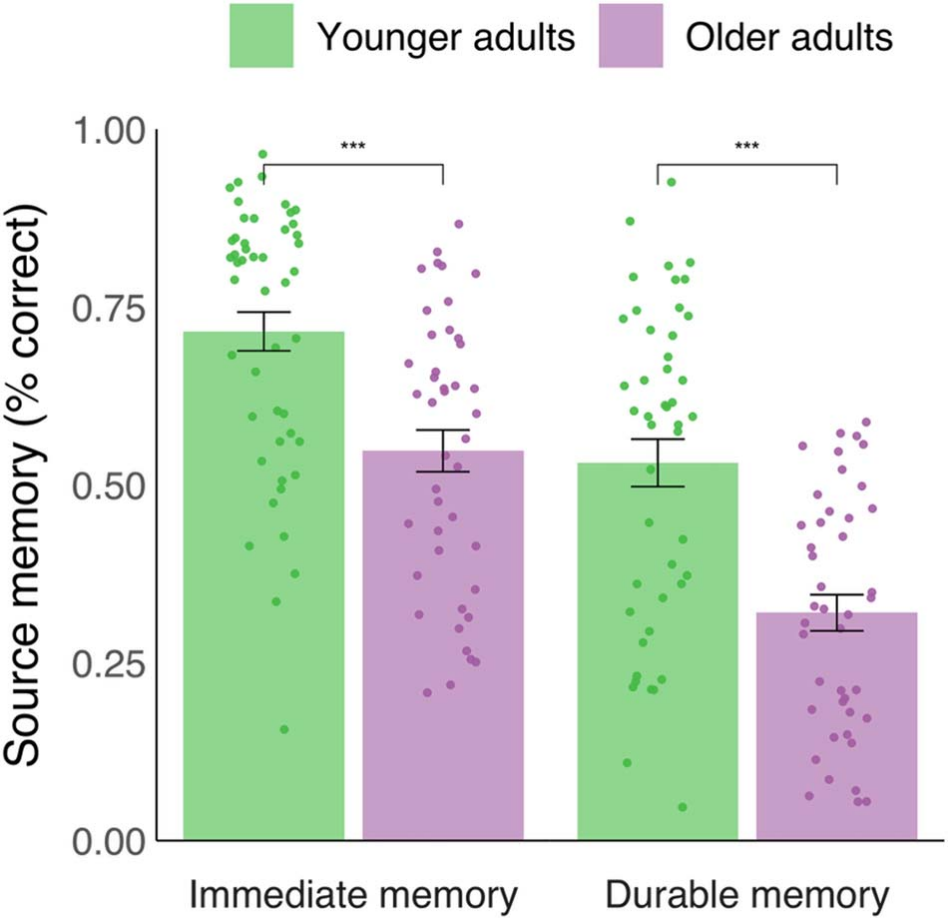
Behavioral analysis. Percentage correct source memory in the immediate and durable memory conditions across age groups (all 91 participants included in analyses). Bar heights represent group means. Dots show data from individual participants. Error bars represent mean ± standard error of the mean. ^*^^*^^*^P < 0.001 (Welch’s independent samples *t*-tests).

We first validated our approach of dividing the sample into two age groups by testing for effects of age on memory performance within the groups. No relationships were observed between age and memory performance among the younger adults (immediate memory: Pearson’s *r* = −0.06, *P* = 0.69; durable memory: *r* = −0.004, *P* = 0.97), or among the older adults (immediate memory: *r* = 0.05, *P* = 0.75; durable memory: *r* = 0.01, *P* = 0.93; see the Supplementary Materials for further tests supporting the differentiation of the sample into two age groups). Percentage correct source memory for each age group is presented in Figure 2. The t-test comparing age groups’ immediate memory performance was significant, showing that older adults performed worse (*M* = 54.76%, SD = 19.25%) than younger adults (*M* = 71.56%, SD = 18.89%) on the short-delay 8AFC test (*t*(87.51) = −4.19, *P* < 0.001, *d* = −0.88). Thus, older adults remembered fewer item-face/place associations than younger adults after a short delay of ~20 min. The t-test comparing age groups’ durable memory performance similarly showed that older adults’ memory performance was significantly lower (*M* = 32.03%, SD = 16.65%) than that of younger adults (*M* = 53.08%, SD = 22.63%; *t*(82.56) = −5.021893, *P* < 0.001, *d* = −1.06). Thus, older adults retained fewer of the initially encoded item-face/place associations than younger adults after an extended delay of ~6 days. This observation was also significant when only including participants with extended delays <9 days (*N* = 85, *P* < 0.001). The ANCOVA revealed that, although immediate memory success was strongly related to durable memory (*F*(1, 86) = 501.90, *P* < 0.001, *η*2 = 0.85), there was a significant effect of age on durable memory performance after controlling for immediate memory (*F*(1, 86) = 3.966, *P* = 0.0495, *η*2 = 0.04), in which older adults showed lower performance (*M* = 39.0%, SD = 14.5%) than younger adults (*M =* 58.1%, SD = 20.0%). Thus, older adults tended to remember fewer item-face/place associations than younger adults after an extended delay of ~6 days even when accounting for the age differences in the immediate memory condition. Excluding four participants with delayed testing intervals >8 days did not change the results (*N* = 85, *P* = 0.03), neither did including test interval as an additional covariate when analyzing the full sample (*P* = 0.011).

### Differences in fMRI Activity for Immediate and Durable Memories, but No Age Effects

**Figure 3:**
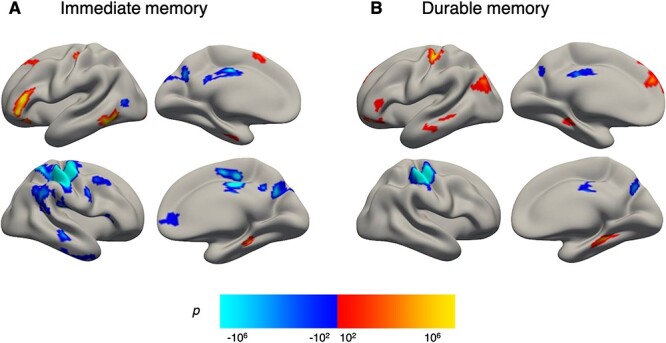
Cortical main effects of encoding activity. BOLD activity during encoding associated with (*A*) immediate memory (immediate memory > miss contrast) and (*B*) durable memory (durable memory > transient memory contrast). Vertex significance is displayed in clusters surviving multiple comparisons correction by FWE (vertex-wise *P* < 0.001; cluster-based *P* < 0.05). Positive and negative significance patterns are shown in respective red–yellow and blue–cyan scales overlaid onto semi-inflated fsaverage5 template surfaces. Abbreviations: BOLD activity, blood-oxygen-level-dependent activity; FWE, family-wise error.

Cortical vertex- and ROI voxel-wise analyses on BOLD signal were performed to identify areas in which activity levels were related to immediate and durable memory performance. Encoding of immediate memories (immediate memory > miss) was associated with increased activity in several left-lateralized prefrontal (superior and inferior gyri), parietal (postcentral gyrus), inferior, and lateral occipital regions, in addition to bilateral fusiform, as well subcortical regions including the left posterior putamen, and the left thalamus (Table 2 and Fig. 3*A*). Activity in the right parietal (postcentral and supramarginal gyri), frontal (inferior frontal and caudal middle frontal gyri), middle- and inferotemporal cortex, bilateral precuneus, posterior cingulate, and lateral occipital cortex, as well as in subcortical regions including right thalamus, was associated with subsequent forgetting (i.e., higher activity in the miss condition).

**Table 2:** Peak activations for (A) immediate memory (immediate memory > miss contrast) and (B) durable memory (durable memory > transient memory contrast) across all participants, with sex as a covariate

Region	Hemisphere	MNI coordinates			Size (mm^2^)	Max (*P*)
		*X*	*Y*	*Z*		
A. Immediate memory > miss						
Pars triangularis	L	−42.7	31.9	6.8	927.40	−5.1656
Inferior temporal	L	−56.2	−51.8	−10.7	700.42	−5.1277
Postcentral	L	−41.0	−19.0	45.3	539.14	−5.5087
Superior frontal	L	−14.9	37.1	42.4	532.80	−4.2204
Lateral orbitofrontal	L	−33.2	29.5	−11.5	357.07	−4.7387
Inferior parietal	L	−31.3	−73.4	34.7	262.38	−4.0045
Fusiform	L	−33.8	−14.6	−26.8	222.38	−4.2982
Lateral occipital	L	−20.0	−97.4	−1.8	181.78	−3.8253
Superior frontal	L	−8.3	17.0	50.7	164.15	−5.1656
Fusiform	R	37.5	−36.0	−12.4	321.45	−4.6199
B. Durable memory > transient memory						
Postcentral	L	−37.0	−26.1	52.4	1129.03	6.5932
Superior frontal	L	−8.6	49.4	23.8	800.13	4.7235
Inferior parietal	L	−43.7	−70.5	29.4	671.58	4.4858
Pars orbitalis	L	−38.1	41.8	−12.5	572.84	5.1687
Pars triangularis	L	−44.6	34.3	2.7	305.46	3.8742
Parahippocampal	L	−28.4	−34.2	−11.9	244.87	4.2892
Middle temporal	L	−63.1	−20.5	−13.4	191.82	3.7019
Middle temporal	L	−57.4	−37.8	−6.1	159.31	3.8450
Parahippocampal	R	35.2	−35.8	−9.1	478.07	5.2237

*Notes:* Left to right columns: the name of the region in which the main peak was localized, the hemisphere, the MNI152 coordinates (*X*, *Y*, *Z*) of the main peaks of the significant clusters (*P* < 0.001), the surface area of the cluster in mm^2^, and the maximum −log10 (*P*-value) in the cluster, FWE-corrected at *P* < 0.05. Abbreviations: L, left; R, Right; FWE, family-wise error.

Encoding of durable memories (durable memory > transient memory) was associated with increased activity in left superior- and inferior frontal, postcentral- and inferior parietal, and middle temporal regions, in addition to bilateral parahippocampal regions, as well as left anterior hippocampus, left caudate, and left putamen. Decreased activity during encoding of durable memories was found in the right parietal cortex and bilateral precuneus (Table 2, and Fig. 3*B*).

As for the effects of age on the memory contrasts, there were no significant differences between younger and older adults in encoding activity levels related to neither immediate memory nor durable memory, as no clusters survived multiple comparison corrections in either contrast.

### Effects of Age on Encoding Connectivity for Durable Memories

**Figure 4:**
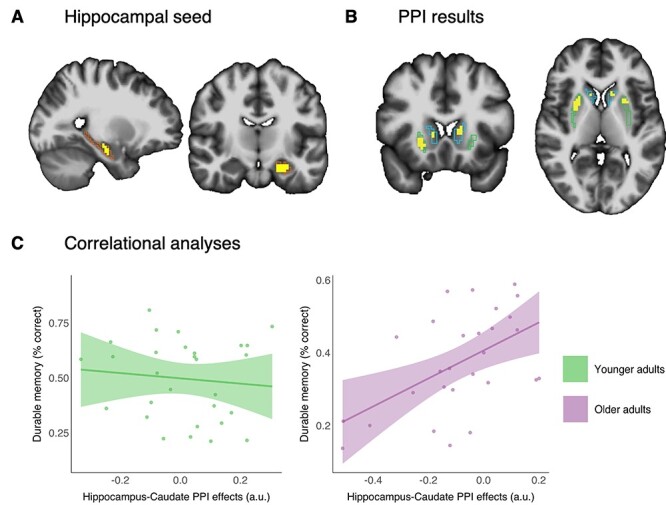
PPI and correlational analyses. (*A*) Sagittal (*X* = 28) and coronal (*Y* = 47) views of the left anterior hippocampal seed in the PPI analysis (hippocampus ROI outline shown in orange, hippocampal seed voxels shown in yellow), identified from the durable memory > transient memory activity contrast. (*B*) Coronal (*Y* = 59) and axial (*Z* = 33) view showing PPI analysis results in which hippocampal FC with bilateral putamen and bilateral caudate was higher in younger compared with older adults during encoding of durable versus transient memories (caudate and putamen ROI outlines shown in blue and green, respectively; significant voxels within the caudate and putamen ROIs shown in yellow; *P* < 0.05, corrected). Both the hippocampal seed region and the significant PPI clusters are overlaid on an MNI152 template brain. (*C*) Relationship between hippocampal–caudate FC (durable memory > transient memory interaction effect) and durable memory performance (% correct source memory) in younger adults (left, green) and older adults (right, pink). Dots show data from individual participants. The straight (green and pink) lines represent the best linear fit to the data and the bands around the line represent a 95% confidence level. Abbreviations: PPI, psychophysiological interactions; FC, functional connectivity; a.u., arbitrary units.

A gPPI analysis was conducted to test whether there was an age difference in FC during encoding of durable versus transient memories. The gPPI compared younger and older adults’ left anterior hippocampal task connectivity to neocortical and dorsal striatal areas during durable versus transient memory encoding. This analysis revealed patterns of stronger connectivity in the younger compared with the older adults between the left anterior hippocampal seed and the bilateral anterior putamen as well as the bilateral middle caudate in the durable memory condition, relative to the transient memory condition (see Fig. 4*B*). Thus, younger adults exhibited greater hippocampal–dorsal striatal FC during encoding of durable versus transient memories than older adults. As for the hippocampal–neocortical effects, some bilateral midline effects were observed in the uncorrected data, suggesting that younger adults’ left anterior hippocampal connectivity with these regions was higher during durable memory encoding compared with older adults. However, none of the surface clusters survived multiple comparison corrections.

Next, in order to assess the relationship between the hippocampal–dorsal striatum connectivity and durable memory performance, robust correlational analyses were carried out between the caudate- and putamen PPI effects and the durable memory scores for both age groups. Results suggested a relationship between higher hippocampal–caudate FC and higher memory performance in the older adults (*r_s_* = 0.0.49, P^*^2 = 0.0156, 2 bivariate outliers; see Fig. 4*C*). No such relationship was observed for the younger adults (*r_s_* = −0.094, P^*^2 ≥ 1, 1 bivariate outlier). The relationship between higher hippocampal–caudate FC and higher memory performance was significantly stronger for the older adults than for the younger adults (*P =* 0.0238). No such relationships were observed between HC–putamen FC and durable memory performance, neither for the older (*r_s_* = 0.26, P^*^2 = 0.37, 2 bivariate outliers) nor for the younger adults (*r_s_* = −0.04, P^*^2 = not significant). Qualitatively similar and significant effects were observed when excluding two participants (one younger and one older adult) with delayed testing interval >8 days: Higher hippocampal–caudate FC was associated with higher memory performance in older adults (*r_s_* = 0.0.54, P^*^2 = 0.006); no significant relationships were observed in younger adults or with the putamen ROI as target.

### Control Analyses

Additional control analyses showed that older adults exhibited significantly higher levels of head motion (*M* = 0.05, SD = 0.04) than the younger adults (*M* = 0.02, SD = 0.02; *t*(58.01) = 3.183795, *P* = 0.002) and that older adults had significantly smaller hippocampal volume (proportion of ICV: Mean (*M*) = 0.0047, SD = 0.0005) compared with the younger adults (*M* = 0.0052, SD = 0.0004; *t*(76.91) = −6.048566, *P* < 0.0001). There was no significant difference in ICV between age groups (older adults, *M* = 1 687 269 mm^3^, SD = 165 131; younger adults, *M* = 1 668 330 mm^3^, SD = 158 238; *t*(83.28) = 0.0547223, *P* = 0.59). Post hoc analyses showed that the observed age group differences in PPI effects between hippocampus and caudate remained significant after controlling for individual differences in head motion, intracortical, and hippocampal volume (*P* = 0.045). The same was true for the observed age group differences in PPI effects between hippocampus and putamen (*P* = 0.049). None of the covariates were themselves predictive of the observed age group difference in PPI effects (all *P* > 0.2). Moreover, the observed positive correlation between durable memory performance and hippocampus–caudate PPI effects in the older adults was also significant after controlling for individual differences in head motion, intracortical, and hippocampal volume (*r_s_* = 0.0.46, P^*^2 = 0.029, 2 bivariate outliers). The correlation between durable memory performance and hippocampus–caudate PPI effects in the younger adults remained not significant, as did correlations between durable memory and hippocampus–putamen PPI effects in both age groups.

Finally, in order to assess whether the observed relationship between PPI effects and behavior (i.e., between hippocampal–caudate FC and durable memory performance in older adults) was independent of univariate activity, we performed two further control analyses. First, robust correlational analyses (Shepherd's pi) between the size of the durable > transient memory contrast effect in the hippocampal ROI and durable memory scores for both age groups showed that there were no significant correlations between hippocampal univariate activity and durable memory, neither for the younger (*r_s_* = 0.026, P^*^2 ≥ 1, 2 bivariate outliers) nor for the older adults (*r_s_* = 0.092, P^*^2 ≥ 1, 3 bivariate outliers). Second, we repeated the robust correlational analysis (Shepherd's pi) between the caudate PPI effect and the durable memory scores while controlling for hippocampal univariate activity (i.e., including the durable > transient memory contrast effect in the hippocampal ROI as a covariate), which showed that there was still a significant relationship between hippocampal–caudate FC and durable memory performance in older adults (*r_s_* = 0.46, P^*^2 = 0.028704, 2 bivariate outliers), while this relationship remained not significant in the younger adults (*r_s_* = −0.13, P^*^2 ≥ 1, 1 bivariate outlier).

## Discussion

We found that older adults tend to have fewer durable memories than younger adults after accounting for immediate memory. While encoding activity did not differ between younger and older adults, older adults showed lower hippocampal–caudate FC during encoding of durable memories. There was also a relationship between higher hippocampal–caudate FC and higher memory performance in the older adults. Together, these results suggest that older adults are characterized by lower hippocampal–striatal connectivity during encoding of durable memories, which could contribute to age-related decline in long-term memory formation.

### Effects of Age on Long-Term Memory Performance

Consistent with existing literature, older adults recalled fewer associations than younger adults after a short delay of 20 min and after a long delay of several days. Moreover, we observed an effect of age on durable memory when controlling for age differences in immediate performance, suggesting that older adults tend to have lower ability to retain memories over longer time periods. Transformation of newly encoded information into long-term storage is a gradual process occurring for weeks and months postencoding, particularly during periods of sleep, which may be disrupted by age-related changes in the structure and function of brain regions important for memory storage (Muehlroth, Rasch, et al. 2020a). Previous research on long-term memory in healthy aging have yielded mixed evidence; some indicate that memory over extended time intervals is relatively more negatively affected by age than memory over shorter time intervals (Wheeler 2000; Davis et al. 2003; MacDonald et al. 2006; Mander et al. 2013; Mary et al. 2013), while others find that forgetting rate is age-invariant (Fjell et al. 2005; Meeter et al. 2005; Vidal-Piñeiro et al. 2017). Notably, the patterns of previous research are reports of either increased forgetting with age or no age differences at all (but see Hulicka and Weiss 1965, for an exception), which suggests that an age-related increase in long-term forgetting exists but can be difficult to identify. Failure to detect consistent age differences could reflect methodological limitations, such as repeated testing of the same material (Vidal-Piñeiro et al. 2017), test format, or task demands such as ceiling effects (Mary et al. 2013). Still, age effects on durable memory tend to be subtle (Elliott et al. 2014).

### Activity during Encoding of Durable Memories

The pattern of activation in left prefrontal, parietal, and bilateral fusiform brain areas during immediate memory success confirms previous research (Kim 2011), except that no hippocampal effect was observed here. Although hippocampal activity is commonly associated with subsequent memory, many studies do not find significant hippocampal or medial temporal lobe effects (for a review, see Henson 2005). It is not clear why the present study did not find hippocampal activation as the use of an associative, pictorial task, presumably prompting elaborative encoding through instructions to vividly imagine an interaction between stimuli would be expected to elicit the medial temporal lobe activity. However, more interestingly, hippocampal activity was associated with durable memory encoding, which is in line with the findings of previous studies (Carr et al. 2010; Wagner et al. 2016). Additionally, activity levels in left prefrontal, parietal, middle temporal, and bilateral parahippocampal regions as well as left caudate were predictive of memory durability, consistent with previous studies (Uncapher and Rugg 2005; Carr et al. 2010; Wagner et al. 2016), along with activity in the left putamen. The subsequent forgetting effects in the right somatosensory cortex can be attributed to vividness ratings. Low vividness items were rated using right-hand buttons and are less likely to be remembered in a later retrieval. The remaining subsequent forgetting effects were roughly found in DMN regions—such as the medial prefrontal, the posteromedial, and the lateral parietal cortices—and thus largely overlap with those reported in previous research (Kim 2011). Subsequent forgetting effects can thus be described as a failure to suppress the DMN (Daselaar et al. 2004) and may reflect either the allocation of processing resources toward internal cognitive events or to reflexive “bottom-up” attention (Wagner and Davachi 2001; Cabeza et al. 2008; Uncapher and Wagner 2009). In both cases, task-irrelevant information is prioritized at the expense of task-relevant information.

We did not observe any differences between younger and older adults in activity related to either immediate or durable memory encoding. This result differs from a previous study in our lab that has examined durable memory in aging (Vidal-Piñeiro et al. 2017). The discrepancy could be due to the use of different encoding tasks, and incidental versus intentional encoding strategies, respectively, although the exact mechanisms are difficult to pinpoint. Nevertheless, the lack of age effects may also suggest that regional activity levels are not the main factor for explaining age-related differences in durable memory formation. For instance, previous research has shown enhanced hippocampal–neocortical FC during encoding of durable memories, in absence of any specific hippocampal activation difference during encoding of durable versus short-term memory (Sneve et al. 2015). This was the rationale behind the focus of the present study on FC during encoding of durable memories.

### FC during Encoding of Durable Memories

Although striatal function has since long been recognized as another important source of age-related cognitive decline (Gabrieli 1996), only recently has research started to uncover a more extensive role for striatum in memory, beyond that of its involvement in a frontostriatal executive network (Nyberg et al. 2016; Rieckmann et al. 2018). Here, we show that compared with young adults, older adults exhibited lower hippocampal–caudate FC during encoding of durable versus transient memories, and a relationship between connectivity and memory performance, which was stronger for older than for younger adults. These results suggest that hippocampal–striatal FC during encoding supports the formation of long-lasting memories and that this mechanism is reduced in aging, which in turn may contribute to the age-related decline in durable episodic memory.

Traditionally, the hippocampus and the striatum have been associated with somewhat different memory processes—a declarative episodic memory system and an incremental reward-based memory system—respectively (Eichenbaum 2004; Shohamy et al. 2008). Animal studies have highlighted a role for the ventral striatum (nucleus accumbens) in reward learning (Roitman et al. 2004) and human studies have demonstrated that the ventral striatum interacts with hippocampus during such learning via dopamine release (Adcock et al. 2006). However, data suggest that also other parts of the striatum interact with hippocampus and that hippocampal–striatal interactions contribute to the formation of single event long-term episodic memories. Studies have shown that dorsal striatal regions and the hippocampus interact during episodic memory encoding (Sadeh et al. 2011) and that the ventral caudate and hippocampus have higher resting state FC in high-performing individuals (Nyberg et al. 2016). Significant overlap in resting state FC patterns for hippocampus and caudate has been observed (Fjell et al. 2016). One study of durable memory formation found a subsequent memory effect in both the left caudate and hippocampus, although no interactions between these structures were reported (Wagner et al. 2016). Similarly, another study found increased activity in both hippocampus and caudate during encoding of memories recognized after a 3-week delay, while this parallel engagement was not observed for immediately recognized memories, suggesting a stronger link between the joint activation of these structures with long-term memory than with immediate memory (Wittmann et al. 2005). These findings collectively suggest that hippocampal and striatal regions are part of a shared functional network that supports the formation of durable episodic memories. This idea is further corroborated by recent demonstrations that the caudate can be functionally differentiated according to its connections with the frontoparietal control network and the DMN and that reduced specificity in coupling with the latter network, including the hippocampus, predicts age-related memory decline (Rieckmann et al. 2018).

The observed relationship between hippocampal–caudate FC and durable episodic memory performance for older adults is in line with Nyberg et al. (2016). These observations may reflect a case of brain maintenance in aging (Nyberg et al. 2012) in which higher-performing older adults have a relatively preserved hippocampal–caudate FC pattern resembling that of younger adults, allowing them to show less age-related memory decline. Moreover, dopamine integrity and hippocampal and caudate volume have all been proposed as indicators of brain maintenance in aging (Nyberg et al. 2012). Future studies utilizing positron emission tomography to map dopamine function could give us a better understanding of how age-related reductions in hippocampal–striatal dopamine and FC relate to each other and to memory decline.

### Limitations

Use of an intentional rather than incidental encoding task could potentially introduce rehearsal effects. Nevertheless, the fairly large number of complex stimuli presented during encoding likely made rehearsal difficult. Also, giving explicit strategy instructions for the encoding task, we gained more control over processing and strategy use than with an incidental encoding task, hopefully minimizing age-related confounds related to differential strategy use (Muehlroth, Sander, et al. 2020b).

A related issue is the potential effects of repeated recall. In addition to the immediate memory test, participants completed an in-scanner retrieval test followed by another 8AFC test approximately 12 h later (data not included here), prior to the long-delay test. This means that the same material was tested three times before the final long-delay memory test and that retrieval processes during the first tests could have enhanced long-term memory performance (note however that the full associative event was only presented once, at encoding, i.e., the correct response was never revealed to the participants). Importantly, younger and older adults seem to equally benefit from prior recall (Wheeler 2000), so that any effect of testing would be less likely to create systematic age differences. Moreover, although postencoding factors during the three preceding memory tests likely influenced performance on the long-delay memory test, we still observed significant differences across age groups in brain activation during transient and durable memory encoding. It is possible that exposure to a small set of face/place stimuli across many trials had negative effects on memory scores, particularly for the older adults. Research has showed that age negatively affects the ability to encode distinct memory representations and that older adults are more susceptible to interference (e.g., Koen and Rugg 2019). As such, repeatedly seeing the same stimuli might have led to more interference and source memory errors for the older than for the younger adults. Future studies could use representational similarity analyses (RSAs) to examine whether representational similarity differences among younger and older adults, especially in cases with repeated stimuli, are related to durable memory performance. Importantly, although the effects of repeated testing are an inherent challenge with the current task design, this feature also allowed us to track each memory’s durability, which would not have been possible without testing the same memories at least twice.

The memory test used in this study has some possible limitations. For instance, because of its forced-choice format and the fact that all the test items had been previously studied, participants did not have the opportunity to indicate whether they did not remember an item altogether. As such, the miss condition likely reflects a mix of trials in which an item was remembered but not its associate as well as trials in which the item itself was not remembered. Furthermore, as the response options in the 8AFC test only included the specific face- and place alternatives (and not general categories of “face” or “place”), it is possible that participants occasionally remembered if an item was paired with face or a place but could not remember which specific face or place. Future studies could divide behavioral responses into more specific categories, such as “source category correct” and “source category incorrect,” which would allow for more fine-grained analyses of behavior.

Adhering to strict criteria for the PPI analysis (i.e., >30 trials in each condition) means that individuals with memory scores in the upper and lower extremes of the data set (about ⅓ of the participants) were not included. The exclusion of high- and low-performing individuals could suggest that our current effect estimates are conservative and implies that future studies aiming to investigate the full behavioral spectrum using PPI will need an even bigger pool of encoded trials than used in the current study (256 trials per participant).

Finally, as the analyzed data in this study were drawn from an extensive project that went over weeks and included multiple phases and tests, some of which were web-based and performed at home, not all participants were able to adhere strictly to our instructions about when to perform the delayed memory test. Observing that the behavioral effects were minimally affected by including participants with longer-than-intended delay intervals, we still performed the main analyses on the full sample; however, all results sensitive to performance on the long-delay memory test were also replicated in a reduced sample characterized by a narrow range of delay intervals between encoding and prolonged retrieval.

## Conclusion

The present study suggests that older adults have a reduced ability to form durable memories. A unique pattern of FC between hippocampus and the caudate contributes specifically to this durable memory formation, which is reduced in older compared with younger adults. These results support and extend the role of striatal connectivity as a potential factor in episodic memory decline in aging. Future studies should examine how postencoding processes such as consolidation and retrieval contribute to memory durability, and how these change with age.

## Supplementary Material

supplementary_materials_bhab331

## Notes

We thank all the participants for their time and effort and our colleagues in LCBC, especially Marie Strømstad, for contributing to data collection. *Conflict of interest*: The authors declare no conflict of interest.
