# Supplementary material for: Reduced Hippocampal-Striatal Interactions during Formation of Durable Episodic Memories in Aging
Source: Cereb Cortex. 2021 Sep 28;32(11):2358–72. doi: 10.1093/cercor/bhab331 (PMC9157302; doi:10.1093/cercor/bhab331)
Supplement: supplementary_materials_bhab331 [file supplementary_materials_bhab331.docx]

**Supplementary materials**

As reported in the main results, no continuous relationships between participant age and memory performance were observed within the two age groups. We also tested for possible non-continuous age effects among the older adults by creating two sub-groups with age ranges 60-70 years (N = 31) and 70-80 years (N = 10). Welch’s t-tests showed no significant differences in source memory accuracy between the two sub-groups (immediate memory: *t*(25.7) = 0.21, *p* = .83; durable memory: *t*(23.7) = 0.42, *p* = .67). Further in line with the behavioral effects presented in the main results, we observed higher memory performance among younger adults both when comparing with the 60-70 years sub-group (immediate memory: *t*(60.1) = 3.58, *p* < .001; durable memory: *t*(73.5) = 4.45, *p* < .001), and with the 70-80 years sub-group (immediate memory: *t*(19.4) = 3.29, *p* = .004; durable memory: *t*(26.3) = 4.19, *p* < .001).

**Table S1**. Number of average trials, ranges, percentiles and sample sizes in each condition of the fMRI univariate- and PPI analyses by age group.

|  | Younger adults | |  |  |  | Older adults | |  |  |
| --- | --- | --- | --- | --- | --- | --- | --- | --- | --- |
|  | Avg. no. of trials | Range | 25^th^/75^th^ pctl. | n |  | Avg. no. of trials | Range | 25^th^/75^th^ pctl. | n |
| A. Univariate analysis conditions | | | | | | | | | |
| Immediate memory | 171 | 43-246 | 130/216 | 47 |  | 133 | 53-221 | 90/168 | 41 |
| Miss | 62 | 9-146 | 33/94 | 47 |  | 104 | 26-191 | 71/144 | 41 |
| Durable memory | 127 | 14-236 | 82/169 | 45 |  | 79 | 9-150 | 47/113 | 40 |
| Transient memory | 47 | 9-110 | 27/65 | 45 |  | 53 | 13-85 | 41/67 | 40 |
| B. PPI analysis conditions | | | | | | | | | |
| Durable memory | 125 | 54-206 | 85/160 | 30 |  | 92 | 35-150 | 62/119 | 30 |
| Transient memory | 59 | 48/69 | 136/210 | 30 |  | 61 | 31-85 | 53/71 | 30 |

Note: The fMRI univariate- and PPI analyses did not include the total sample of 91 participants as exclusion criteria for these analyses were stricter than for behavioral analyses. For univariate analyses (A), participants with < 8 trials in any of the conditions and/or with a mean accuracy below 25% on the short-delay 8AFC test were excluded. For the PPI analysis (B), participants with less than 30 trials in either of the two relevant conditions were excluded (see Methods for more details on the exclusion criteria and the samples included the univariate- and PPI analyses). Abbreviations: Avg., average; no., number; pctl., percentile; n, sample size; PPI, psychophysiological interactions.
